# Role of lateral soft tissue release in percutaneous hallux valgus surgery: a systematic review and meta-analysis of the literature

**DOI:** 10.1007/s00402-022-04693-x

**Published:** 2022-11-10

**Authors:** Antonio Izzo, Salvatore Vallefuoco, Morena Anna Basso, Robbie Ray, Francesco Smeraglia, Andrea Cozzolino, Massimo Mariconda, Alessio Bernasconi

**Affiliations:** 1 Department of Public Health, Trauma and Orthopaedics, University of Naples Federico II, Naples, Italy; 2 King’s Foot and Ankle Unit, King’s College Hospital NHS Foundation Trust, London, UK

**Keywords:** Hallux valgus, Percutaneous, Minimally invasive, Lateral release, Meta-analysis

## Abstract

**Background:**

It is unclear whether lateral soft tissue release (LSTR) is required as part of percutaneous hallux valgus (PHV) surgery. The primary aim of this systematic review was to assess whether LSTR reduces the risk of recurrence of hallux valgus deformity. The secondary aims were to assess if LSTR increases the risk of complications, improves the clinical outcome and leads to a greater radiographic correction.

**Methods:**

We performed a PRISMA-compliant PROSPERO-registered systematic review, pooling clinical papers reporting results after PHV surgery into two categories (PHV with (Group 1, G1) and without LSTR (Group 2, G2)) and comparing them. Data regarding the study design, demographics, the surgical procedure and the clinical and radiological outcome were extracted and compared. Risk of bias was assessed using the modified Coleman Methodology Score (mCMS).

**Results:**

Sixteen studies were selected (G1:594 feet; G2:553 feet). The pooled proportion of recurrence at a minimum 21-month follow-up (2%, 95%CI 0–3 vs 2%, 95%CI 0–5; *p* = 0.70) did not differ in the two groups. Similarly, the pooled proportion of complications (27%, 95%CI 17–38 vs 25%, 95%CI 12–37; *p* = 0.79) was similar. The pre- (*p* = 0.23) and post-operative AOFAS scores (*p* = 0.16), the pre-(HVA: *p* = 0.23) (IMA: *p* = 0.94) and post-operative radiological angles (HVA: *p* = 0.47) (IMA: *p* = 0.2) and the methodological quality of studies (*p* = 0.2) did not differ either between G1 and G2.

**Conclusion:**

There is no evidence that LSTR performed during percutaneous HV surgery reduces the risk of recurrence of the deformity at a mean 4-year follow-up nor improves the clinical and radiological outcome.

**Level of evidence:**

Level IV systematic review of Level I to IV studies.

## Introduction

Hallux valgus (HV) is one of the most common forefoot disorders encountered in orthopaedic clinics. Its prevalence has been estimated at around 23% in adults aged 18–65 years [1] In symptomatic cases, surgical correction is indicated with the aim to realign the first ray and relieve the patient from symptoms [2]. Multiple techniques have been reported with a satisfactory outcome, with no proof of superiority of one technique over another [2]

The most common approach for mild to moderate HV generally involves a distal metaphyseal or metaphyso-diaphyseal osteotomy of the first metatarsal aimed to shift the metatarsal head laterally. The value of a simultaneous derotation of the metatarsal in the coronal plane (to correct the excessive pronation) and in the transversal plane (to restore the distal metaphyseal metatarsal angle or DMMA) has been discussed over recent years and is now considered a key element to reduce the risk of recurrence of the deformity [3–6] In case of residual interphalangeal valgus, a closing wedge osteotomy of the proximal phalanx (i.e., Akin osteotomy) is often recommended [7, 8] The use of percutaneous approaches to perform such osteotomies is increasing over time, with an increasing number of studies documenting the non-inferiority as compared to open techniques and overall good results [9–13]

With regard to soft tissues, the increased tension of lateral structures such as the abductor tendon of the hallux, the joint capsule, the lateral sesamoid suspensory ligament and the lateral collateral ligament has been well described in anatomical studies [14–16] and their release (lateral soft tissue release or LSTR) has been advocated from some authors as paramount to achieve a satisfactory alignment and reduce the risk or recurrence of the condition [3, 17, 18]. Two main types of percutaneous LSTR have been described, i.e. the isolated adductor tenotomy and the combined percutaneous lateral release [19] In a narrative review of the literature, Del Vecchio et al. have highlighted that different investigators prefer to perform different types of release and that in many studies it is unclear which structures are being released [19] Additionally, in a cadaveric study by Dalmau Pastor et al. a case of FHL rupture after LSTR was documented, which raises concerns about potential additional risks of LSTR which yet need to be quantified [16]

With this background, we performed a systematic review of the literature aimed to compare the results of percutaneous hallux valgus (PHV) surgery with and without LSTR to assess the difference in recurrence rate at medium term follow-up. The secondary aims of the study were to evaluate whether LSTR increases the risk of complications, improves the clinical outcome and leads to a greater radiographic correction in PHV.

## Methods

### Protocol and registration

This systematic review was designed according to the Preferred Reporting Items for Systematic reviews and Meta-Analyses (PRISMA). It was prospectively registered in the PROSPERO database (CRD42022304574).

### Eligibility criteria

To be included, all the following criteria had to be met: studies reporting data after PHV in patients aged between 15 and 85 years; clear description of the surgical technique with one or more statements about LSTR (homogeneous series in which LSTR was systematically performed or systematically not-performed); minimum follow-up of 6 months; assessment of clinical results through pre- and post-operative dedicated scores; radiographic assessment of pre- and post-operative angles on weightbearing standardized radiographs; randomized, quasi-randomized, prospective and retrospective cohort studies, case series; published in English, Spanish, Portuguese, French and Italian; full text availability either online either after direct contact with the authors.

Exclusion criteria were as folows: studies reporting results after open surgery; studies not reporting surgical details or not performing distal or diaphyseal osteotomies; studies on proximal osteotomies or Lapidus procedure; data on skeletally immature patients; case reports, technical notes, biomechanical studies, cadaveric studies, expert opinions, letters to the editor, studies on animals and instructional courses. Narrative or systematic reviews were also excluded from the study but references were double checked in order to identify potential eligible studies.

### Information sources and search

A systematic search was conducted on Pubmed, Embase, Cochrane Library and Scopus, from the earliest entries through October 22, 2021 with the following Boolean operators: ((hallux valgus) OR (bunion) OR (hallux abduct*)) AND ((percutaneous) OR (minimally invasive) OR (miniopen)). Additional studies were identified in the bibliographies of these articles. Two reviewers (AI and DM) independently screened the results of the research; then full text of eligible studies were analyzed. Disputes were resolved by the senior author (AB).

### Data charting and items

Data were charted independently by two investigators (SV and MB) using an Excel sheet. Results were compared to verify that no data were missed. Data extracted were as follows: year of publication, type of study, level of evidence, demographics (sample size, sex, age), type of surgery, additional procedures, length of follow-up, clinical scores with pre- and post-operative values, pre- and post-operative radiographic angles (hallux valgus angle or HVA and intermetatarsal angle or IMA), postoperative complications and recurrences. Based on the aim of the study, studies were pooled in two groups: PHV with LSTR (Group 1 or G1) and PHV without LSTR (Group 2 or G2). For studies reporting results of different techniques in different series, each series was analysed independently.

### Risk of bias

The modified Coleman Methodology Score (mCMS) was used to assess the quality of studies included, ranging from 0 to 100, as already done in prior foot and ankle literature [20–22]. Two investigators performed the mCMS assessment twice (AI and DM), with an interval of 10 days, and discussed the scores when more than a two point difference was present, until consensus was reached. A score higher than 85 was considered excellent, good from 70 to 84, fair from 50 to 69 and poor when less than 50, as reported previously [22].

### Synthesis of results

Baseline data in the two groups were tested for normality using a Shapiro–Wilk test. A proportional meta-analysis was run to pool data regarding the recurrence and complication rate. Although all studies had a minimum follow-up of 6 months, only studies reporting a follow-up longer than 21 months were considered to assess the recurrence rate. The ‘metaprop’ command was used to compute 95% confidence intervals using the score statistic and the exact binomial method and incorporate the Freeman–Tukey double arcsine transformation of proportions. Heterogeneity among studies was assessed through the Higgins’ *I*^2^ statistic and a random-effect model was applied in all cases. A meta regression was used to compare pooled proportions between the two groups. Pooled clinical scores and radiographic angles were obtained as average value and reported along with the standard deviation (SD) and range values. Normally and non-normally distributed continuous variables were compared between the two groups using a Student *T*-test or a Wilcoxon rank sum test, respectively. Categorical data were compared using a *χ*^2^ test. The significance level for the overall estimates of effect was set at *p* < 0.05. All analyses were performed using STATA statistical software package (Version 14.0, StataCorp, 2015).

## Results

### Studies included

**Fig. 1:**
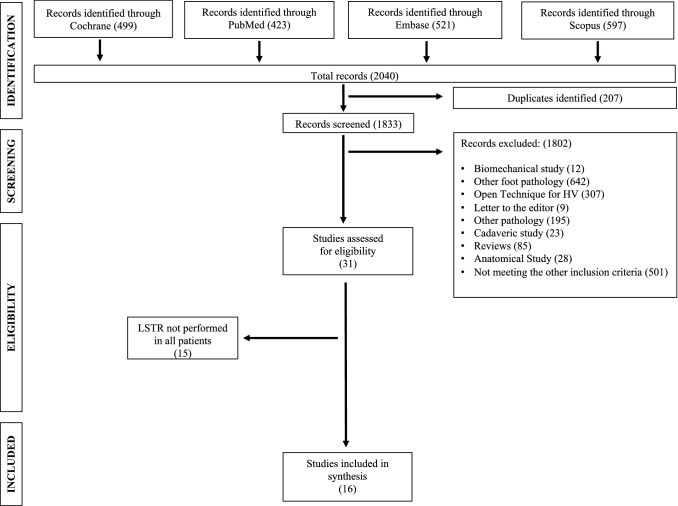
Flow chart for studies included in this systematic review

Out of 1833 studies, 16 were selected including 1147 feet in 960 patient (594 feet in G1 and 553 feet in G2) (Fig. 1) [10, 11, 13, 23–35] In one study two series (two techniques) were included (Table 1) [30] The sample size (*p* = 0.11), sex distribution (*p* = 0.57), the length of follow-up (*p* = 0.79) and the size of the incision (*p* = 0.13) were comparable. Studies in G2 included a younger population (54 ± 6.3 vs 44.4 ± 6.8 years; *p* = 0.006); however, age did not correlate with the clinical and radiological outcome (all *p* > 0.05) (Table 2). The methodological quality of studies (mCMS: 68.7 ± 11 points in G1, 63.4 ± 14.3 points in G2; *p* = 0.2) was similar in G1 and G2 (Table 1).

**Table 1:** Main characteristics of studies included in this review

Author (year)	Study design	LoE	Technique	Incision (cm)	Akin (Y/N)	Concomitant procedures (Y/N)	Definition of release	mCMS	Sample size (feet)	Follow-up (m)
With LSTR										
Barragan-Hervella (2008) et al. [23]	Prosp—Non comp	IV	Isham-De Prado	1	Yes	No	Adductor Tenotomy, Lateral Caps	43	29	6
Lai (2018) et al. [10]	Prosp—Comp	III	Chevron	0.3	Yes	Yes	DSTR	60	29	24
Crespo-Romero et al. (2018) [24]	Prosp—Non Comp	II	Isham—De Prado	1	Yes	Yes	Lateral Metatarsal Arthrolysis	71	132	57.3
Kaufmann et al. (2019) [8, 11]	RCT	I	Chevron	0.5	Yes	No	LSTR	87	25	9
Liuni et al. (2020) [25]	Prosp—Non Comp	IV	PBS	0.5	Yes	No	Adductor Tenotomy, Lateral Caps	73	58	25
Maniglio et al. (2019) [26]	Retr—Non Comp	IV	Bösch	0.5	No	Yes	LSTR	65	30	12.6
Severyns et al. (2019) [27]	Retr—Non Comp	IV	Reverdin-Isham	0.3	Yes	Yes	Lateral Caps, Ligament Release	77	57	60.1
Kaufmann et al. (2020) [28]	Prosp—Comp	I	Chevron	0.5	No	No	LSTR	75	19	67.1
Del Vecchio et al. (2021) [29]	Prosp—Non Comp	IV	Chevron	0.3	Yes	No	Adductor Release	78	114	24
Marijuschkin et al. (2021) [30]	Prosp—Comp	IV	Isham-De Prado	1	Yes	Yes	LSTR	66	36	17.2
Marijuschkin et al. (2021) [30]	Prosp—Comp	IV	Chevron	1	Yes	Yes	LSTR	66	35	17.2
Torrent et al. (2021) [13]	RCT	I	Scarf	0.5	Yes	Yes	MI Lateral Release	64	30	21
Without LSTR										
Valles-Figueroa et al. (2010) [31]	Retr—Non Comp	IV	Bösch	0.3	No	No	No	43	40	6
Sun et al. (2010) [32]	Retr—Non Comp	IV	Bösch	1	No	Yes	No	61	150	90
Radwan and Mansour (2012) [33]	Retr—Comp	IV	Bösch	1.5	No	No	No	67	31	21.7
Faour-Martín et al. (2013) [34]	Prosp—Non Comp	IV	Bösch	1	No	No	No	83	115	121
Siddiqui et al. (2021) [35]	Retr—Comp	IV	Bösch	1	No	Yes	No	63	217	9.3

*LSTR* lateral soft tissue release, *Prosp* prospective, *Retr* retrospective, *Comp* comparative, *non-comp* non comparative, *RCT* randomised controlled trial, *LoE* level of evidence, *mCMS* modified Coleman Methodology Score, *DSTR* distal soft tissue release, *Y/N* yes/not, *Lateral Caps* lateral capsulectomy, *MI* minimally invasive, *PBS* percutaneous Bianchi system, *m* months

**Table 2:** Baseline characteristics of the cohorts investigated in the studies included in this review

	With LSTR		Without LSTR		*p* value
	Mean ± SD	Range	Mean ± SD	Range	
Feet (N)	49.5 ± 36.5	19–132	110.6 ± 77.8	31–217	0.06
Patients (N)	45.4 ± 31.3	19–132	83 ± 59.6	84.2–90.2	0.11
Age (y***)***	54.0 ± 6.3	4264	44.5 ± 6.8	32.7–49	0.006
Sex (%F)	0.9 ± 0.07	0.7–1	0.7 ± 0.3	0.1–0.9	0.57
Follow-up (m)	28.4 ± 20.9	6–67.1	49.6 ± 52.5	6–121	0.79
Size of incision (cm)	0.6 ± 0.3	0.3–0.1	0.9 ± 0.4	0.3–1.5	0.13

*LSTR* lateral soft tissue release, *SD* standard deviation, *N* number, *F* female, *y* years, *m* months, *cm* centimeter

### Recurrence rate

**Fig. 2:**
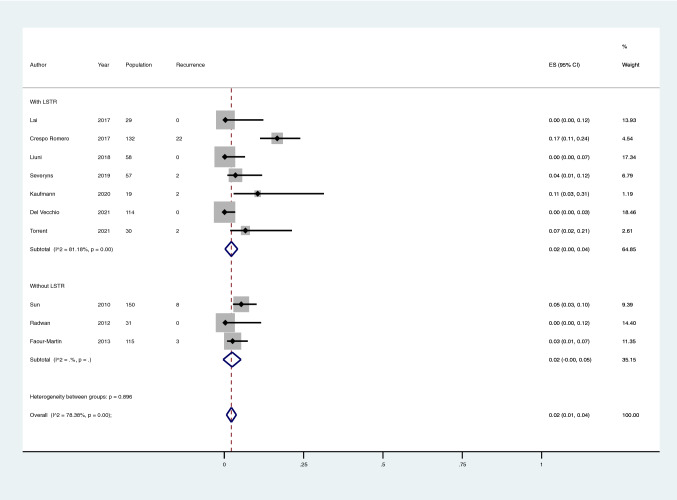
Meta-analysis of the proportion of recurrence of Hallux Valgus in patients that have undergone percutaneous Hallux Valgus surgery with and without lateral soft tissue release. Output generated by the Stata procedure *metaprop*

The pooled proportion of recurrence at a minimum 21-month follow-up (mean follow-up: 51 months) was 2% (95%CI 0–3) in G1 and 2% (95%CI 0–5) in G2, without a statistical difference (*p* = 0.70) (Fig. 2). The intra-group heterogeneity was substantial in G1 (*I*^2^ 68.3%; *p* < 0.001) but not significant in G2 (42.9%; *p* = 0.14) (Fig. 2).

### Complication rate

**Fig. 3:**
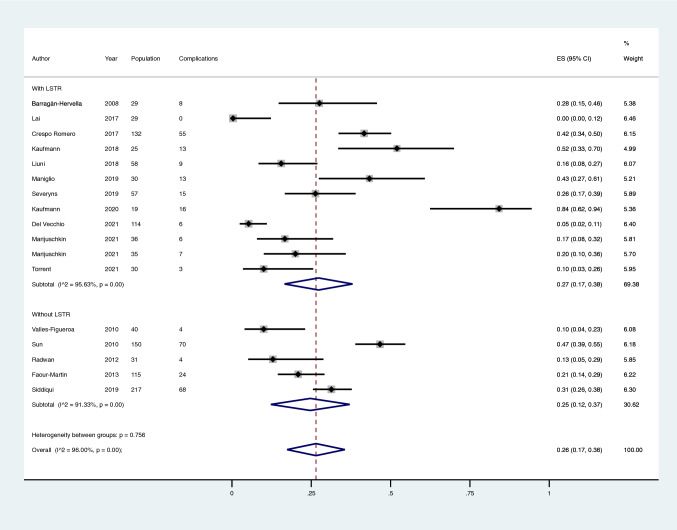
Meta-analysis of the proportion of complications in patients that underwent percutaneous Hallux Valgus surgery with and without lateral soft tissue release. Output generated by the Stata procedure *metaprop*

The pooled proportion of complications in the two groups (G1: 27%; 95%CI 17–38 and G2: 25%; 95%CI 12–37, respectively) was not significantly different (*p* = 0.79) (Fig. 3; Table 3). The intra-group heterogeneity was considerable both in G1 (*I*^2^ 95.6%; *p* < 0.001) and G2 (91.3%; *p* < 0.001) (Fig. 3).

**Table 3:** List of complications extracted from the studies included in this review

Authors (year)	Complications (including recurrence)
With LSTR	
Barragan-Hervella (2008) et al. [23]	8 (27.5%) o 2 (7%) persistent oedema o 4 (13%) persistent pain o 2 (7.5%) wound dehiescence
Lai (2018) et al. [10]	No Complication reported
Crespo-Romero et al. (2018) [24]	55 (41.6%) o 9 (6.8%) superficial infection o 4 (3%) stiffness o 3 (2.3%) reflex sympathetic distrophia o 3 (2.3%) neuromas o after dmmo: 2/61 (3.2%) pseudoarthrosis o after dmmo 5/61 (8.1%) transfer metatarsalgia o after dmmo 7/61 (11.4%) recurrence metatarsalgia o 22 (16.7%) recurrence (medial pain)
Kaufmann et al. (2019) [8, 11]	13 (52%) o 12 (48%) soft-tissue irritation caused by the Kirschner wire o 1 (4%) recurrence (‘mild recurrence of deformity’)
Liuni et al. (2020) [25]	9 (15%) o 5 (8.6%) metatarsalgia o 3 (5.1%) severe stiffness 5.1% o 1 (1.7%) persistent paresthesia
Maniglio et al. (2019) [26]	13 (30%) o 2 (4%) nonunion o 4 (10%) metatarsalgia o 1 (2%) metatarsal stress fracture o 1 (2%) secondary toe deformity o 3 (7%) early K-wire removal due to soft-tissue inflammation o 2 (4%) recurrence
Severyns et al. (2019) [27]	15 (26.3%) o 4 (7%) transfer metatarsalgia o 1 (1.7%) deep vein thrombosis o 2 (3.5%) recurrence o 5 (8.7%) wound dehiescence o 3 (5.2%) self-resolving paresthesia
Kaufmann et al. (2020) [28]	16 (84%) o 16 (84%) soft-tissue irritation caused by the Kirschner wire (of which 2 (10%) recurrence)
Del Vecchio et al. (2021) [29]	6 (5.2%) o 1 (0.8%) transfer metatarsalgia o 3 (2.6%) soft tissue irritation o 1 (0.8%) superficial infection
Marijuschkin et al. (2021) [30]	6 (17.6%) *Isham technique* o 3 (8.3%) joint stiffness o 3 (8.3%) symptomatic callus 7 (20%) *Chevron technique* o 3 (8%) irritation from metalwork o 1 (3%) fistula o 1 (3%) joint stiffness o 2 (5%) transfer metatarsalgia
Torrent et al. (2021) [13]	3 (10%) o 1 (3.3%) skin irritation o 2 (6.6%) recurrence
Without LSTR	
Valles-Figueroa et al. (2010) [31]	10 (25%) o 2 (12.5%) persistent pain o 2 (12.5%) recurrence of the deformity
Sun et al. (2010) [32]	70 (46%) o 62 (41%) transfer metatarsalgia o 8 (5%) recurrence (persistent valgus)
Radwan and Mansour (2012) [33]	4 (14%) o 2 (6.9%) pin infection o 2 (6.9%) joint stiffness
Faour-Martín et al. (2013) [34]	24 (20%) o 3 (2.6%) skin irritation o 2 (1.7%) deep infections o 16 (13.9%) joint stiffness o 3 (2.6%) recurrence (> 25° for HVA)
Siddiqui et al. (2021) [35]	68 (30.6%) o 42 (19.4%) pin site infection o 8 (3.7%) nerve-related numbness o 6 (2.8%) hardware failure o 3 (1.4%) asymptomatic malunion o 3 (1.4%) delayed union o 1 (0.5%) sesamoiditis o 5 (2.3%) bunion recurrence

*Dmmo* distal metatarsal metaphyseal osteotomy, *HVA* Hallux Valgus Angle

### Clinical and radiographic outcome

The pre- (G1: 51.7 ± 10.6 and G2: 45.8 ± 1.7 points; *p* = 0.23) and post-operative AOFAS scores (G1: 89.4 ± 4.3 and G2: 86.9 ± 3.2 points; *p* = 0.16) and the pre- (HVA: G1: 29.7 ± 2.9 and G2: 44.1° ± 26.8°; *p* = 0.23) (IMA: G1: 12.5 ± 4.2 and G2: 14.1° ± 2.6°; *p* = 0.94) and post-operative radiological angles (HVA: G1: 12.1 ± 4.3 and G2: 12.3° ± 2.3°; *p* = 0.47) (IMA: G1: 9.2 ± 2.2 and G2: 7.9° ± 1.3°; *p* = 0.2) did not differ in the two groups (Table 4).

**Table 4:** Clinical scores and radiographic angles at the last follow-up reported in the two groups

	With LSTR		Without LSTR		*p *value
	Mean ± SD	Range	Mean ± SD	Range	
Pre AOFAS (points)*	51.7 ± 10.6	28.6–65	45.8 ± 1.8	44.6–47.1	0.23
Post AOFAS (points)*	89.4 ± 4. 3	84–96.6	87 ± 3.2	84.2–90.2	0.16
Pre IMA (degrees)*	12.6 ± 4.2	0–15.3	14.1 ± 2.6	11.7–17.6	0.94
Post IMA (degrees)*	9.3 ± 2.3	6–12.6	7.9 ± 1.3	4.7–9.1	0.2
Pre HVA (degrees)*	29.7 ± 2.9	26.4–34.3	44.1 ± 26.8	27.6–84.2	0.23
Post HVA (degrees)*	12.2 ± 4.4	6.9–22.5	12.3 ± 2.3	8.4–14.6	0.47

*LSTR* lateral soft tissue release, *SD* standard deviation, *AOFAS* American Orthopaedic Foot and Ankle Society score, *IMA* intermetarsal angle, *HVA* hallux valgus angle

^*^reported in 15/17 series, i.e. studies by Barragan-Hervella [23] and Valles-Figueroa [31] did not report AOFAS values while studies by Valles-Figueroa [31] and Siddiqui et al. [35] did not report radiographic measurement

### Other confounding factors

Although Akin osteotomy was performed more frequently in G1 (*p* = 0.001), it did not correlate with the clinical or radiographic outcome (all *p* > 0.05). The proportion of studies in which a concomitant procedure was reported along with the PHV was not different in the two groups (*p* = 0.32).

## Discussion

The main finding of this systematic review was that the release of lateral soft tissues during percutaneous correction of Hallux Valgus does not seem to influence the recurrence rate at a mean 4-year follow-up. Also, the number of complications occurring in these patients is similar both whether the release is performed or not. Additionally, the clinical outcome (assessed through the AOFAS score) and the radiographic correction of the deformity at the longest follow-up did not differ in the two groups. Overall, the quality of studies was only fair both for series in which LSTR was performed or not.

Upon review of the literature, the value of LSTR still appears debated. In a recent meta-analysis, Yammine et al. investigated six comparative studies dealing with the open Chevron osteotomy performed with and without LSTR [36] The authors concluded there was a beneficial effect deriving from the transection of the lateral sesamoid metatarsal ligament on the hallux valgus angle in all types of deformity (from mild to severe), also suggesting a possible efficacy of adductor transection in moderate deformities and a benefit from trans-metatarsal ligament transection in severe hallux valgus [36] Interestingly, the authors documented a twofold risk of hallux varus in the LSTR group as compared to the non-LSTR one (2% vs 0.95%), albeit the difference did not achieve statistical significance in their cohort [36] While it is difficult to infer about why our findings did not support the hypothesis that LSTR leads to a greater correction after PHV surgery, it has been suggested that percutaneous techniques may allow a greater lateral shift of the metatarsal head during the procedure, maximizing the medialization of the proximal fragment and subsequentially increasing the transversal stability of the first tarsometatarsal joint [37] Although this could theoretically play a role in reducing the recurrence rate regardless of LSTR, we acknowledge that such hypothesis would require further studies in order to be confirmed. A second theory which could reasonably explain the similar recurrence rate in the two groups is that the correct realignment of the axis of the first ray may efficiently neutralize the valgus deforming soft tissue forces, bringing them back to their stabilizing role. In other words, while it is clear that, once the physiological axis of the first ray has deviated, the progressive contraction of lateral soft tissues contributes to maintain and worsen the deformity, it is still unknown whether they may play a role as a trigger of HV [3] As a reminder, in normal feet these structures are physiologically counterbalanced by medial soft tissues and act as static and dynamic stabilizers of the joint. So the question arises whether after restoring a straight axis of the first ray there is a real need to lengthen lateral tissues.

On a different note, we found significant heterogeneity of definitions applied to LSTR from different authors (Table 1) and confirmed the lack of standardization already claimed in previous studies [3] In two-thirds of studies selected in this review, only a generic description of the surgical procedure such as ‘distal soft tissue release’ or ‘capsuloligamentary release’ was adopted, which does not clarify the structures targeted, limiting reproducibility and inter-study comparisons. Noteworthy, we had to exclude studies where the authors performed the LSTR only depending on the intraoperative correctability of the deformity (before or after the osteotomy) and without providing in the results a distinction between those who underwent LSTR and those who did not. Although several studies in cadavers have provided a detailed description of the positioning of tendons and ligaments relative to the joint capsule and sesamoids [14–16], we found that most authors apply arbitrary criteria as to when to perform the LSTR. This experience- rather than evidence-based practice affects any attempt to clarify the role of LSTR in the final the outcome of PHV surgery, keeping the question ‘is LSTR needed?’ still unanswered.

In terms of complications, we expected to find a greater number of iatrogenic hallux varus in the LSTR group, but this was not the case. Even if it is ascertained that iatrogenic hallux varus is related to the overcorrection of both soft tissues and bony structures [38–40] one could argue that the integrity of the lateral collateral ligament and the underlying joint capsule might somehow protect from an excessive varus deviation of the hallux. While the literature reports an incidence between 2 and 13% for iatrogenic hallux varus [38, 40] quite surprisingly in this cohort of 1147 HVs this complication never occurred. We fear that this discrepancy could derive from a reporting bias in primary studies, which unfortunately hinders drawing a reliable conclusion regarding LSTR and the risk of hallux varus. With regard to LSTR-specific complications, none were clearly reported in the studies selected. We also hypothesized that some of the nerve-related complications (i.e., numbness, paresthesia, neuromata) documented by authors could be secondary to an injury of the terminal branch of the deep peroneal nerve [15] but data provided was not sufficient to verify or disprove this conclusion.

There were multiple limitations to this study. The level of evidence provided by our analysis is IV due to the inclusion of Level I to IV studies. This was due to the paucity of prospective comparative studies on LSTR, which are advocated in the future. The heterogeneity in the description of LSTR between studies is another important limitation of this study, which reflects the different surgical habits adopted in different centers around the world. The quality of studies included, as demonstrated through the mCMS, was only fair which negatively affects the strength of our findings. Although we ran a proportional meta-analysis on the main variable of interest, we could only compare the average values of clinical scores and radiographic angles, which weakens our findings about these variables. Most studies adopted only the AOFAS score as measurement of the clinical status which is not validated, limiting the analysis of clinical outcomes. The statistical heterogeneity reported for the complication rate was significant in both groups analysed, which might add a further bias to our results. Finally, although the mid-term results in terms of recurrence after PHV are certainly of interest for the orthopaedic surgeon, it would be advisable to repeat our analysis in a few years when the primary studies will allow to assess the recurrence rate at a longer follow-up.

## Conclusion

In this systematic review, we found no evidence that lateral soft tissue release performed during percutaneous hallux valgus surgery reduces the risk of recurrence of the deformity at a mean 4-year follow-up nor improves the clinical and radiological outcome. Further prospective comparative studies are advocated to shed some more light in this area.

## Data Availability

Data from this study can be made available by authors upon request.
